# Uncovering compound heterozygous *DYSF* variants in a Chinese family affected by limb-girdle muscular dystrophy type 2B

**DOI:** 10.3389/fgene.2025.1664086

**Published:** 2025-09-17

**Authors:** Jinlan Li, Jie Zhou, Chunbo Ji, Siqing Ma, Jianying Zhu, Tiejun Yang, Danyang Dong, Yang Ping

**Affiliations:** ^1^ Clinical Medicine School of Ningxia Medical University, Yinchuan, China; ^2^ Department of Neurology, General Hospital of Ningxia Medical University, Yinchuan, China

**Keywords:** variants, DYSF, dysferlin, limb-girdle recessive muscular dystrophy 2, limb-girdle muscular dystrophy type 2B

## Abstract

This case concerns a Chinese female patient who was referred to our clinic having complained of weakness in her lower limbs. Following a series of diagnostic procedures, including electrophysiology, muscle biopsy and genetic analysis, the patient was diagnosed with limb-girdle muscular dystrophy type 2B (LGMD2B). Genetic testing revealed compound heterozygous mutations in the *DYSF* gene, specifically the missense mutation c.6313G>A (p.Ala2105Thr). Another variant, c.4444del (p.Glu1482Serfs*43), is a frameshift mutation. This case provides further confirmation of the LGMD2B diagnosis. It also identifies novel compound heterozygous *DYSF* mutations. These findings have significant implications for the diagnosis and research of genetic diseases, the management of at-risk individuals and the development of new therapies.

## Introduction

Limb-girdle muscular dystrophy (LGMD) is a hereditary disorder marked by progressive skeletal muscle atrophy and weakness due to gene mutations. In the Chinese population, LGMD2B, caused by *DYSF* gene mutations, and LGMD2A, linked to CAPN3 mutations, account for 65.9% of cases (Lin et al., 2023). LGMD2B primarily impacts hip and shoulder muscles and is associated with the absence or dysfunction of dysferlin, a protein crucial for muscle cell membrane repair (Bouchard, 2023). This report describes the clinical case of a female patient who presented with a progressive weakness in both of her lower limbs. Initially misdiagnosed with polymyositis due to persistently elevated serum creatine kinase (CK) levels, she was eventually diagnosed with LGMD2B following muscle biopsy and genetic testing, including next-generation sequencing (NGS). Sanger sequencing revealed compound heterozygous mutations in the *DYSF* gene. These mutations have not been previously reported in the literature, indicating their rarity. This case highlights the importance of an accurate diagnosis in hereditary neuromuscular diseases and encourages further research into the genetic mechanisms that underlie LGMD.

## Case presentation

Here we present a case of a 38-year-old unmarried woman from a kinship family in north-west China who has suffered progressive limb wasting for 23 years. Her clinical history is as follows: The patient was admitted to our hospital’s neurology department in 2001 at the age of 15 due to “bilateral lower limb weakness that had lasted for 2 years.” At that time, she could not stand up quickly after squatting and gradually lost the ability to coordinate walking and jumping. A neurological examination revealed no abnormalities in the cranial nerves. Bilateral lower limb muscle strength (including the pelvis and quadriceps primarily) was rated as grade 4 (maximum 5 points) and distal muscle strength was rated as grade 5. Both Babinski signs were negative, while the Gowers sign was positive.

Laboratory results showed serum creatine kinase levels of 27,990.83 μmol/L. Electrophysiological tests revealed chronic myogenic damage in both lower limbs. EMG showed no spontaneous potentials at rest and revealed a significantly shortened mean motor unit duration, as well as an increased number of polyphasic waves during light exercise. Polymyositis was diagnosed. The patient received intravenous methylprednisolone therapy, resulting in symptom relief and restoration of ambulation. Following treatment, the creatine kinase level fell to 3.423.7 μmol/L, after which the patient was discharged.

However, the patient was readmitted in 2005 for persistent stair-climbing weakness, having been treated with methotrexate and folic acid. Electrophysiology and a muscle biopsy confirmed damage to the gastrocnemius muscle with atrophy, hypertrophy, and fatty hyperplasia, which is consistent with LGMD 2B. Until 2024, the patient remained untreated for LGMD 2B, which resulted in severe disability and total dependence on a wheelchair. An echocardiogram showed no abnormalities, while laboratory tests revealed irregularities in the enzymes. Genetic testing clarified the subtype of limb-girdle muscular dystrophy and its genetic characteristics.

**FIGURE 1:**
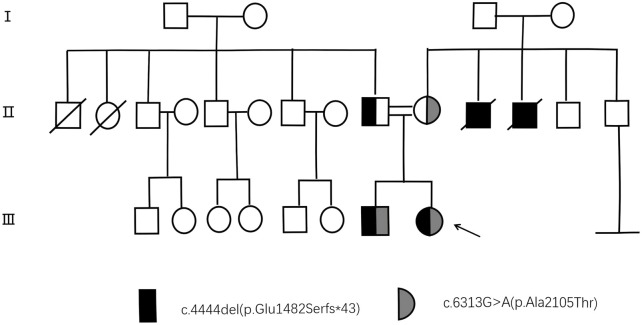
Pedigree chart illustrating genetic inheritance in the Patient’s family.

Prior to venipuncture, informed consent was obtained from the patient, her parents and control subjects. Although the patient’s parents appeared phenotypically normal, there were concerns about their consanguinity due to a family history of genetic diseases (two uncles became ill at around 20 years of age and later died in their 40s). To illustrate the genetic inheritance among the patient and her family members, a pedigree has been shown in Figure 1. (This pedigree chart is a display of the genetic inheritance of the patient and her family members. Individuals carrying the mutation are shown in black. Individuals without the mutation are shown in white. Squares represent male relatives, circles female relatives and arrows the proband).

**FIGURE 2:**
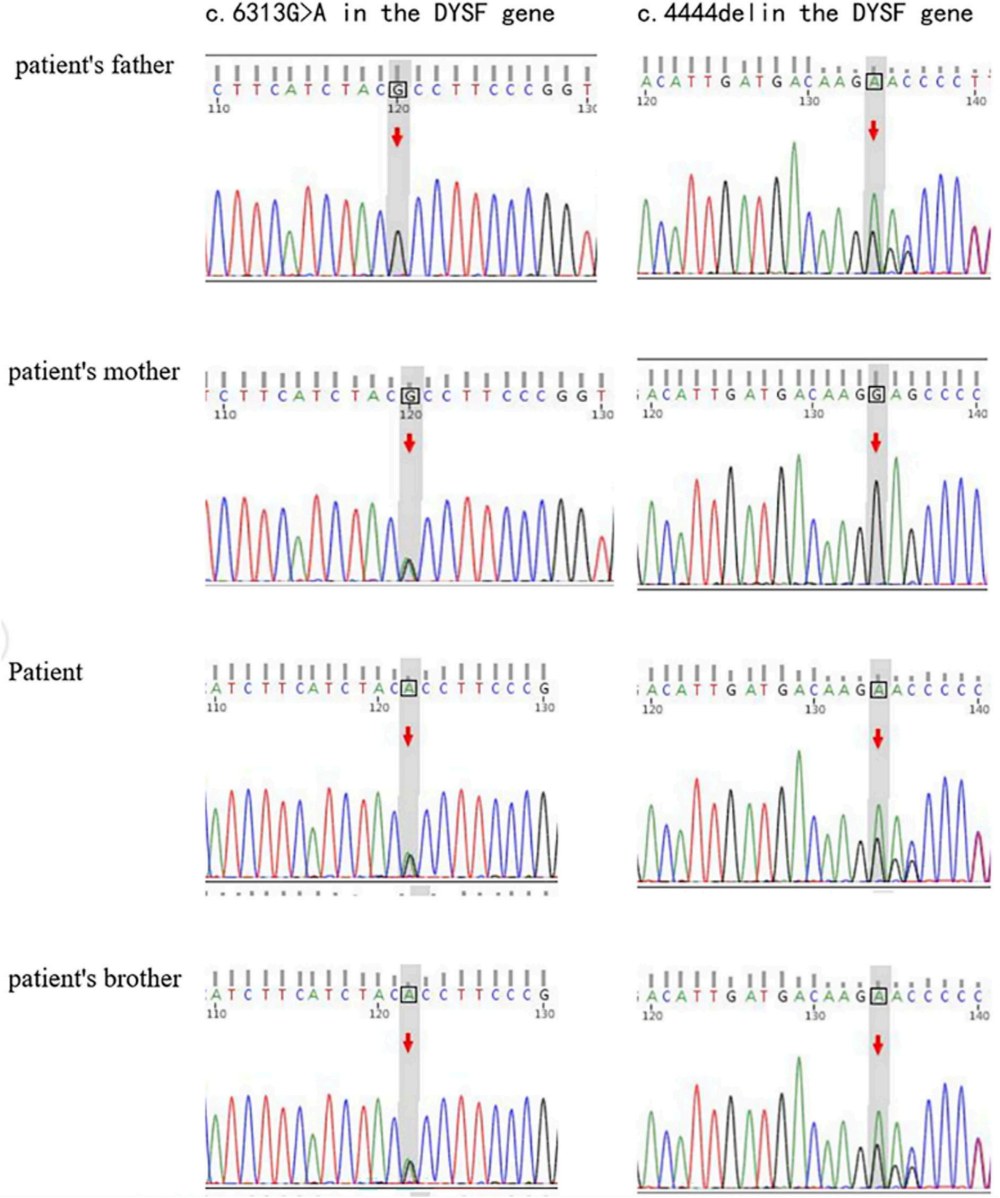
Genetic mutations in the *DYSF* gene. Sanger sequencing plot showing *DYSF* variable sequences in *DYSF* gene affected families.

Genetic testing shown in Figure 2 has revealed that the individual carries the variant c.6313G>A (p.Ala2105Thr), a missense mutation in the *DYSF* gene affecting codon 2015. The mutation has been confirmed by Sanger sequencing and is associated with Miyoshi dystrophy/limb-girdle dystrophy, with three heterozygotes reported in the gnomAD database. Another variant is a frameshift mutation caused by a three nucleotide deletion inherited from the father, c.4444 del (p.Glu1482Serfs*43). It shows potential pathogenicity, although it is classified as a variant of uncertain significance (VUS) according to the ACMG guidelines. A novel compound heterozygous variant in the *DYSF* gene was identified by genetic sequencing, confirming the diagnosis of LGMD2B.

## Discussion

Muscle biopsies in most patients with ferritinopathies show an inflammatory response, particularly pronounced with neurological abnormalities (Angelini et al., 2018), This suggests that inflammation may manifest relatively early, as symptomatic conditions can occur even in patients exhibiting fewer clinical signs. This suggests inflammation may present early, as symptoms can arise even in patients with fewer clinical signs (Rowin et al., 1999). Additionally, dysferlin deficiency activates inflammatory vesicles in muscle, creating a pro-inflammatory environment that worsens damage (Rawat et al., 2010). Consequently, misdiagnosis of LGMD as polymyositis due to the inflammatory response of the muscle is prevalent in the early stages of the disease, and genetic testing is a pivotal discriminating factor. Such misdiagnosis can lead to decreased muscle strength, reduced bone density, and other adverse effects (Nguyen et al., 2005). Recent studies show that intermittent prednisone administration can reduce muscle damage without causing atrophy, but its impact on disease progression remains unconfirmed (Quattrocelli et al., 2017; Zelikovich et al., 2022).

We compared two mutation sites in the *DYSF* gene using the public databases dbSNP and ClinVar and identified the mutation c.6313G>A (p.Ala2105Thr, referred to as mtB). Three heterozygous cases were reported in the gnomAD database, but no homozygous cases were found. The literature indicates that the mtB variant is present in patients with Miyoshi myopathy and limb-girdle muscular dystrophy, often in homozygous or compound heterozygous forms (PMID: 32400077, 27066573, 21522182, 34559919, 32528171, 30028523). Based on current evidence, the clinical significance of this variant is uncertain. In contrast, the c.4444 del (p.Glu1482Serfs*43, designated mtA) variant has not been reported in the literature and is absent in gnomAD. Based on the available data, this variant is thought to be pathogenic.

**FIGURE 3:**
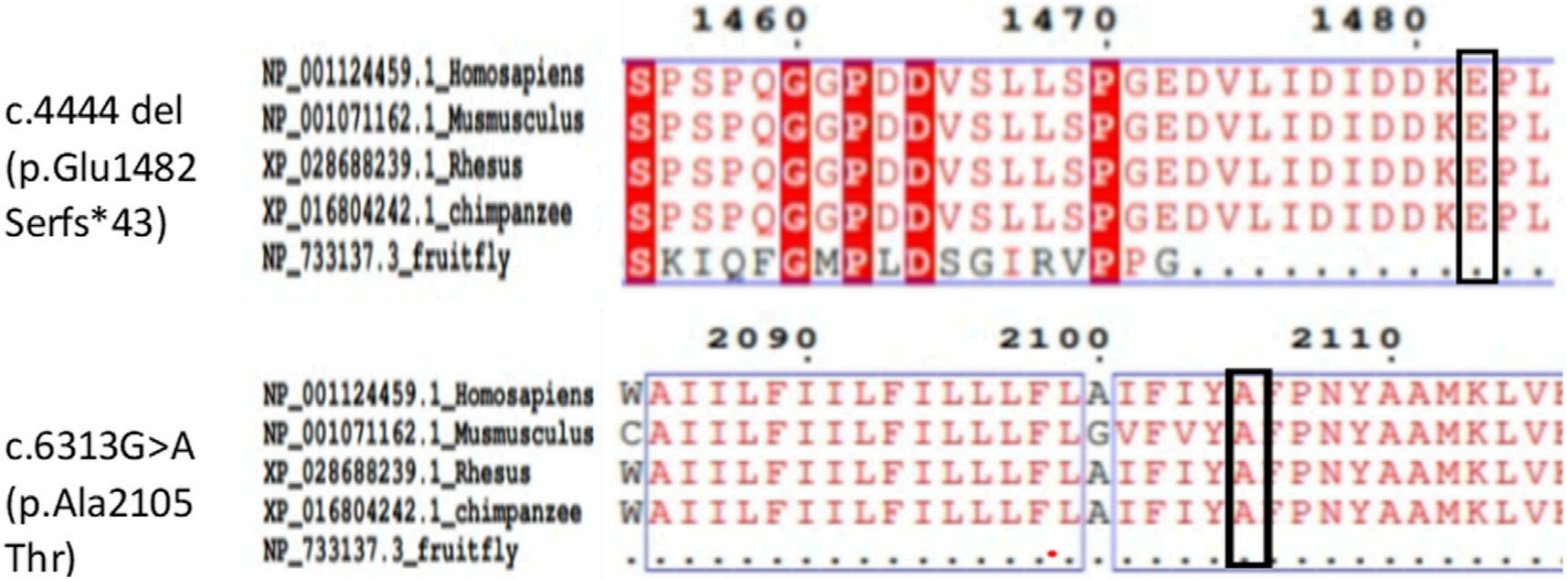
Amino acid sequence conservation analysis of DYSF proteins of different species.

Amino acid sequences of the DYSF protein from *Homo sapiens*, *Mus musculus*, rhesus monkey, chimpanzee and *Drosophila melanogaster* were obtained from the NCBI database. Alignments performed with Clustal Omega (https://www.ebi.ac.uk/Tools/msa/clustalo/) showed a high degree of conservation of amino acid sites corresponding to these mutations (Figure 3).

**FIGURE 4:**
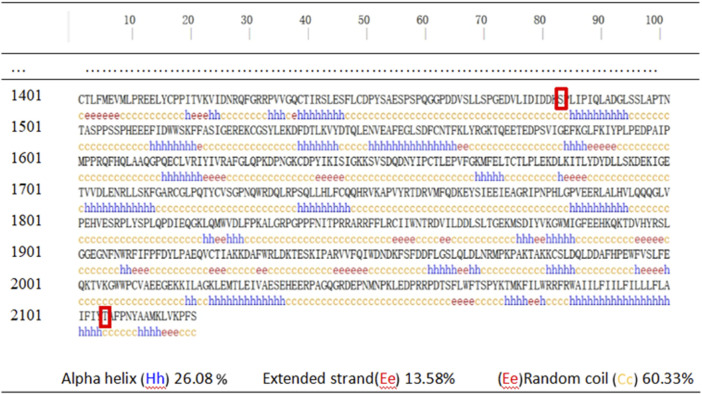
Schematic representation of the secondary structure of DYSF protein.

**FIGURE 5:**
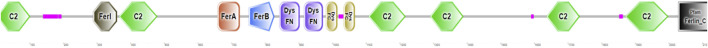
Protein structural domains of DYSF.

The secondary structure of human DYSF was predicted using PSIPRED v4.0 and showed that the affected amino acids were located within irregular coil structures (Figure 4). Domain predictions from the Pfam and SMART databases indicated that mtA is outside the main domain, while mtB is within the Ferlin_C domain (Figure 5).

**FIGURE 6:**
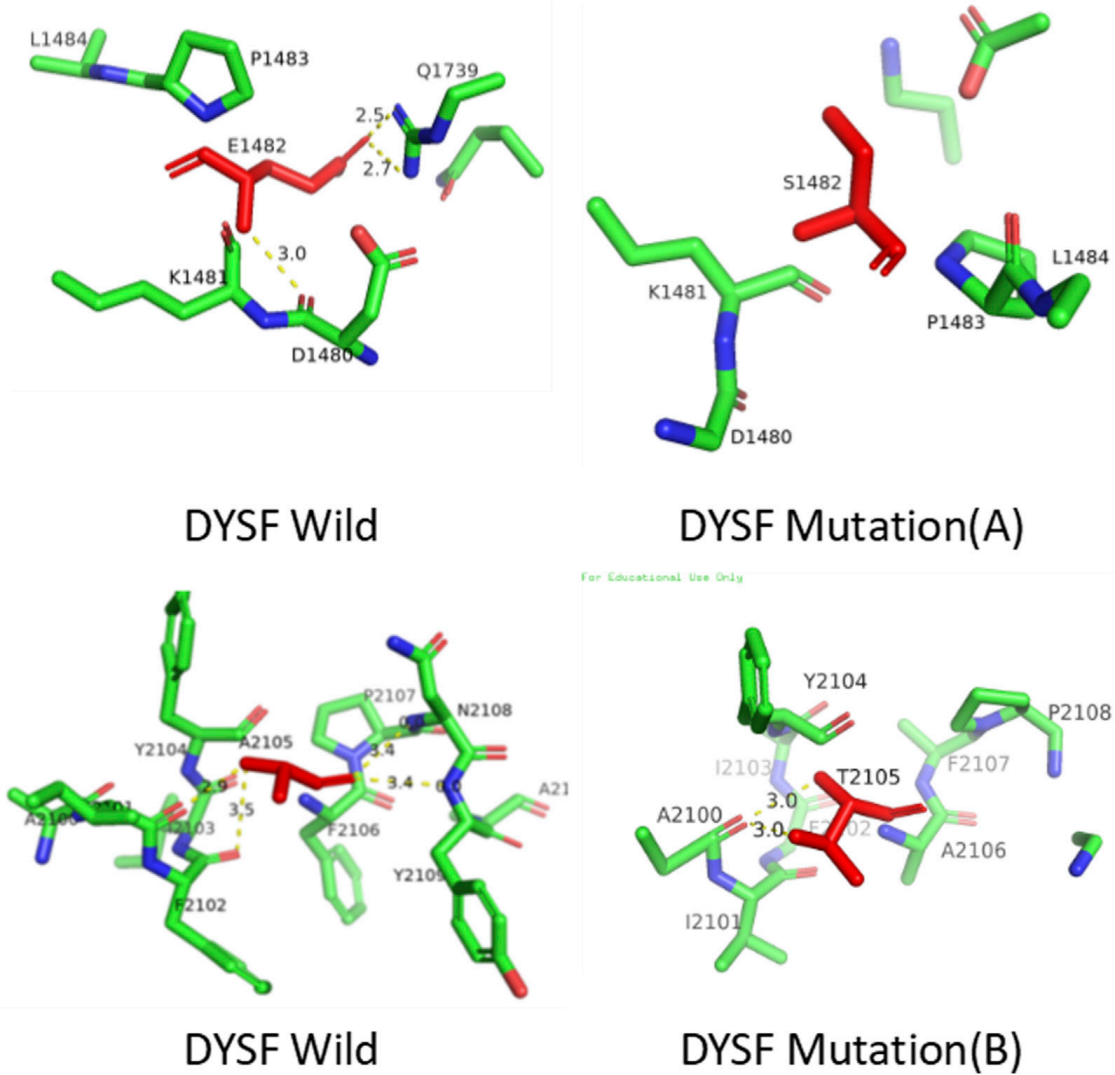
Schematic representation of the tertiary structure of wild type and mutant human DYSF.

Three-dimensional structures of the wild-type and mutant proteins were constructed using SWISS-MODEL (http://swissmodel.expasy.org/). Analysis revealed that mt A replaces glutamic acid with serine, resulting in the loss of three hydrogen bonds. Pathogenicity of the DYSF gene: c.4444 deletion mutation (p.Glu1482Serfs*43, mtA) variant. This mutation is not located in the key functional domain or active site of the DYSF protein (as shown in Figure 5), so it may not affect enzyme activity or ligand binding. However, it may indirectly affect function by altering protein stability or interaction interfaces (as depicted in Figure 6). Therefore, we conclude that the observed phenotype in the proband is most likely caused by a frameshift mutation leading to protein truncation. while, mt B replaces alanine with threonine, resulting in the loss of one hydrogen bonds (Figure 6). These mutations change the structure of the protein from an ordered to a disordered state, affecting its functionality.

This report details a significant case of limb-girdle muscular dystrophy type 2B (LGMD2B) in a female patient, emphasising the crucial diagnostic steps involved, such as muscle biopsy, advanced gene sequencing, and bioinformatics analysis. The discovery of previously unreported DYSF mutations not only facilitates further investigation into their pathogenicity but also broadens our understanding of hereditary neuromuscular diseases. By exploring the genetic complexities of these disorders, we enhance the potential for earlier diagnosis and improved patient management.

## Data Availability

The datasets presented in this article are not readily available because of ethical and privacy restrictions. Requests to access the datasets should be directed to the corresponding author.
